# Editorial

**Published:** 1850-04

**Authors:** 


### LATERAL CAVITY PLATES.

We see in the last number of the American Journal and Library of Dental Science, an article on this subject, by Dr. Dwinelle, one of its editors, which, from its practical character, is well worth a careful perusal. We have within the last two years renewed our experiments on plates with air chambers, and although we have never attached the spring spoken of and described by Dr. Dwinelle, yet the lateral cavity plates have received a due share of attention, and we confess with more success than efforts which we made some eight or ten years since: by reference to the first volume of the Dental Register, p. 229, it will be seen that we alluded to this subject in July, 1848. We have found two or three things as essential in the success in all suction plates, or indeed in plates where no suction is intended or aimed at. First, the plate should be well adapted to the mouth, and pressure made on one side or in front, should not occasion the plate to fall from the gums at any point. No set of teeth can be of much service in masticating unless this is accomplished.

Second. This being obtained, the plate is ready for the addition of chambers; and we have found that unless the adaptation around the border of the chamber was perfect, they are of but little avail. When both is accomplished, either the central cavity or the lateral cavities will secure a more firm retention of the plate. Our aim is always, first, to secure the suction of a plain plate. If this, after repeated efforts, appears impracticable, then we try the central or lateral cavities. Yet, of a great many sets of teeth which we have inserted on the atmospheric principle, those now giving the most satisfaction are free from any chamber. We are, however, determined, as soon as possible, to get entirely rid of clasps for the attachment of artificial teeth. This subject we shall resume again.—[J. T., Cin. Ed.

### THE ST. LOUIS PROBE.

THIS is the name of a new medical monthly, the first number of which was issued in the first month of the present year, and bids fair to *Probe* to the quick all deviations from that professional etiquette, which is truly the cause of so much wrangling among medical practitioners. The medical profession, in every department, (the dental as well as any other,) is interested in the sustaining of such a work, and we hope before the first half year expires, a sufficient number of subscribers may be obtained to justify the making it a bi-weekly, as contemplated.

We see from the prospectus, that it is the only "medical periodical" which has been issued in St. Louis since May last. The Missouri Medical and Surgical Journal and the St. Louis Medical and Surgical Journal, we hope are not both discontinued ; yet we see as one of the editors of the Probe, a name rendered familiar by its association with the former.

The Probe is published monthly, by Drs. Coons & Atkinson, "each number containing one sheet," at $2 per annum, payable in advance.—[J. T., CIN. ED.

### CONTRIBUTIONS.

WE would tender our thanks to the members of the Mississippi Valley Association of Dental Surgeons, for the *very prompt* manner in which they have sent in their contributions. They will excuse us, however, for the non-appearance of their articles in the present number of the Register. We will promise, however, that all that *has been sent in*, or that *may be in*, in time for the next number, will be forthcoming in our next.—[J. T., CIN. ED.

### OHIO COLLEGE OF DENTAL SURGERY.

THIS Institution closed its fifth annual course the last of February. Five young gentlemen were examined, and received the degree of Doctor of Dental Surgery. These were :

| | |
|---|---|
| JOSIAH RAMSAY, | Springfield, O. |
| WM. H. KING, | Pittsburgh, Pa. |
| JONATHAN TAFT, | Xenia, O. |
| J. W. KEYES, | Florida. |
| H. S. CHENOWETH, | Circleville, O. |

The regular course of instruction commences the first Monday of every November.—[J. T., CIN. ED.

